# Does persistence make you healthy? An empirical study on female entrepreneurs from China

**DOI:** 10.1186/s12905-021-01471-6

**Published:** 2021-09-08

**Authors:** Heqi Jia, Zhengda Xu, Song Lin, Feng Jiang

**Affiliations:** 1 Business School, Central University of Finance and Economics, Beijing, 100081 China; 2 Business School, Beijing Technology and Business University, Beijing, 100048 China

**Keywords:** Female entrepreneur, Health, Entrepreneurial experience, Firm age

## Abstract

**Background:**

Nowadays, more and more women are engaging in entrepreneurial activities. Meanwhile, female entrepreneurs’ health problems have been increasingly reported worldwide. What factors would influence female entrepreneurs’ health are the subject of this paper.

**Methods:**

This paper focuses on the effects of entrepreneurial experience and age of firm on female entrepreneurs’ health through the analysis of 2 years of tracking data in the Bohai Economic Rim, which is one of the most developed areas for entrepreneurial activities in China.

**Results:**

Results from the samples of female entrepreneurs demonstrate that increasing entrepreneurial experience and growing firm age could help female entrepreneurs to activate multiple positive identities. These identities can help female entrepreneurs cope with gender stereotype threat and maintain good health.

**Conclusion:**

This paper contributes to entrepreneur health research in two aspects. First, this study focused on entrepreneurial history indexed by entrepreneurial experience and firm age, enriching the field of female entrepreneurship. Second, this study further explored the mechanism that women cope with stereotype threat in the context of entrepreneurship. At the same time, this paper addresses ways that policy-makers and social media are responsible to help female entrepreneurs stay healthy.

**Supplementary Information:**

The online version contains supplementary material available at 10.1186/s12905-021-01471-6.

## Background

Nowadays, more and more women are engaging in entrepreneurial activities, resulting in similar amounts of female and male entrepreneurs. Meanwhile, female entrepreneurs’ health problems have been increasingly reported worldwide. For example, Liu Qing, the president of DiDi (Chinese version of Uber), a famous female leader in China, was diagnosed with breast cancer which was highly associated with entrepreneurial stress in 2015.^1^ In addition, the founder of Kate Spade suicided due to the bipolar disorder in 2018.^2^ We have to notice that female entrepreneurs are facing severe health problems during the Covid-19 pandemic. BDC (Business Development Company) reported that female entrepreneurs (51%) were suffering more depression than male entrepreneurs (40%) under the pandemic context.^3^

When a novel virus (Covid-19) outbreak in 2019, the economy and society face great uncertainty [1]. The ventures were forced to close down to avoid virus spread. It influenced the firm's general operation, such as production and sales [2]. The cash flow of many ventures was facing severe challenges [1]. Entrepreneurs also suffer great psychological distress due to the uncertainty and unpredictable economic environment, especially for female entrepreneurs [3]. Keeping good health is beneficial to female's quality of life during the Covid-19 pandemic [4]. However, the health problems of female entrepreneurs have been largely ignored in empirical studies. Finding what factors would influence female entrepreneurs' health is a significant topic to help them overcome the tough times' challenges. It is also the subject of this article under the pandemic context.

The study of entrepreneurial health has become a frontier research field in entrepreneurship [5]. Some of scholars found that female entrepreneurs were less healthy than male entrepreneurs because they were affected more by working pressure [6], but others found argued that female entrepreneurs were less likely to suffer from cardiovascular disease than male entrepreneurs [7]. Meanwhile, some other empirical findings suggest no gender difference in health status of entrepreneurs [8, 9]. The research of female entrepreneurs’ health is deserved to be investigated.

Currently, scholars have examined the factors which would affect entrepreneurs’ health. Some scholars found entrepreneurs were more likely to resist the positive effects of health [10, 11]. On the other hand, aggressive and hostile entrepreneurs were opted to suffer more health problems [8, 12].

In addition, the uniqueness of entrepreneurship as a career choice also impacts on entrepreneurs’ health conditions. Many studies find that as entrepreneurial practices proceed, entrepreneurs will face stress from several aspects such as market uncertainty [13] and financial uncertainty [14], and then sacrifice personal health. From another perspective, entrepreneurship also has advantages for health: the fact that entrepreneurs enjoy great autonomy and gain psychological benefits by being their own bosses is good for health [15]. The pressure starts when they choose to launch a business and become entrepreneurs [16]. Because they not only have to be responsible for the strategy, action, and success of a company but also have to take care of satisfying their shareholders, friends and families [10]. Entrepreneurial pressure would lead to entrepreneurs with anxiety and deep depression, and bad physical health [10].

Compared to male entrepreneurs, female entrepreneurs may hold lower entrepreneurial intention [17], report lower opportunity evaluation [18], and have more difficulties to obtain entrepreneurial finance [19]. Female entrepreneurs are suffering the negative stereotype threat because they are not consistent with the feminine social role [20–23]. This stereotype threat will bring lots of symptoms, such as increases in arterial blood pressure [24], heart rate variability [25], electroencephalogram [20], cardiovascular arousal [26]. These symptoms are belonging to physical health. Physical health refers to the physiologic and physical status of the body [27].

There are still some research gaps of current studies. First, persistence is vital for entrepreneurs to achieve success [28]. There exists sex differences in entrepreneurship [29]. Scholars have not discussed whether female entrepreneurs’ persistence can help them to maintain health. Second, scholars have verified the entrepreneurial experience significantly influence entrepreneurs’ psychological well-being [16]. However, scholars neglected that entrepreneurial experience as a persistence identity would also influence entrepreneurs’ physical health. Third, firm age is a survival duration variable [30]. It also represents a persistence identity for firms. The existing research lacks the analysis of the impact of enterprise characteristics on the health of female entrepreneurs. Fourth, the physical health of entrepreneurs is neglected by scholars in entrepreneurship [31], especially for female entrepreneurs. Thus, we just focus on how persistence identity influence female entrepreneurs’ physical health in this paper.

Our paper uses stereotype threat theory to explain which factors would be beneficial to female entrepreneurs’ physical health. We find that female entrepreneurs activate the identity of experienced entrepreneurs as they have much more entrepreneurial experience and activate the identity of successful entrepreneurs as the firm age increasing. These positive identities are helpful for female entrepreneurs to overcome gender stereotype threat and maintain good health.

## Theory and hypothesis

### Gender stereotype threat in entrepreneurship

Gender stereotypes reflect shared beliefs about specific characteristics of men and women; for example, men perform better than in math than women [17, 32, 33]. In the business world, women find themselves in a stigmatized group as well [34]. The entrepreneur role is often characterized as bold, risk-taking, and aggressive [18]. Men are regarded as more agentic, and women are regarded as more communal [35, 36]. Entrepreneurs are thought to have more masculine than feminine characteristics [22]. Thus, female entrepreneurs suffer from stereotype threat because female entrepreneurs are not consistent with feminine perceived social role, which leads to negative judgment [20–23]. The stereotypes they faced may create a disruptive state that undermines women’s professional performance and aspirations [37]. When stereotypes have a negative impact on groups, it is called stereotype threat [38].

The Covid-19 pandemic challenges people's life [4]. Due to the virus spread rapidly, governors gave the "stay-at-home" orders to try to slow down the spread [39]. The Covid-19 pandemic lockdown changed the world people are living in. Females are considered to put much time into taking care of their family, especially their children, not their career. Thus, female entrepreneurs suffered more gender stereotype threats under the pandemic context [40].

Gender stereotype threat can negatively influence the entrepreneurial process for women [33, 41]. Additionally, stereotype threat can affect biological factors [42]. Stereotype threat could cause a series of neurobiological consequences, such as increasing in arterial blood pressure [24], heart rate variability [25], and cardiovascular arousal [26], as well as causing stress due to unfair expectations [20, 24]. Thus, female entrepreneurs expose to stereotype threat might experience negative health consequences [37].

### Intervening in stereotype threat

Stereotype threat intervention is a valuable way to help individuals get over stereotype threat [38]. Previous psychological literature has shown that holding a positive personal identity helps distinguish oneself from the stereotyped group [43, 44]. Identities are ongoing projects arising from dialogues between an inner self and an external discourse reflecting the social domain [22].

When women do not perceive themselves to be targeted by stereotypes, they may avoid stereotype threat [43, 44]. When female entrepreneurs have more entrepreneurial experience, they become more confident and regard themselves as experienced entrepreneurs [22]. As firms established by female entrepreneurs grow older, their founders believe in themselves more and consider themselves successful entrepreneurs [45, 46]. Female entrepreneurs who establish a positive identity may avoid stereotype threat, leading to a better health status.

### The influence of entrepreneurial experience

Generally, entrepreneurship is considered as an agentic activity [18, 47]. However, as entrepreneurial experience increases, female entrepreneurs can obtain transferable skills and competencies from previous start-up experiences [22]. Prior start-up experience is defined as an individual's past experience relating to previous creation or founding of new business ventures [16]. Experienced entrepreneur sends the signals to external stakeholders that female entrepreneurs are credible and capable in entrepreneurial activities [48–50]. External stakeholders may believe in female entrepreneurs’ ability rather than continuing undervaluing the role of female entrepreneur due to gender stereotype [33]. Consequently, when female entrepreneurs are labeled as experienced entrepreneurs, they can build this positive identity which can effectively eliminate the stereotype threat faced by female entrepreneurs.

When female entrepreneurs become serial entrepreneurs, it shows that they have self-evaluated as a “capable entrepreneur”. Such women can also learn from failure and apply their knowledge to current business practices [51]. Only those entrepreneurs who believe that they are smart enough will start businesses again after failure [52]. Even though female entrepreneurs have experienced stereotype threat in past entrepreneurial activities, the success of those activities, which turns out to be a psychological self-affirmation may help them overcome stereotype threat [53].

Thus, with the entrepreneurial experiences increasing, women can identify as experienced entrepreneurs, which in turn can improve their health. The following hypothesis is therefore proposed:

#### **H1**

 Among female entrepreneurs, the previous entrepreneurial experiences are positively related to their physical health.

### The influence of firm age

Firm age can be regarded as the firm experience which gains from the entrepreneurial activities [54]. Aged firm is beneficial to reduce gender stereotype threat as well. Increased firm age is an important indicator to measure whether a firm is successful [46]. Because start-ups come with higher risks, many new ventures unfortunately end in failure [52, 55]. As firm age increases, the likelihood of entrepreneurial failure decreases [55, 56]. Female entrepreneurs in such firms are then regarded as successful entrepreneurs.

Female entrepreneurs also acquire greater knowledge about how to manage a successful business during the process of entrepreneurship [57]. This process teaches women to compete and survive in their industry [46]. When female entrepreneurs are able to identify themselves as people with multiple positive traits, they are less susceptible to gender stereotype threat [44].

Therefore, due to firm age growth, women can identify themselves as successful entrepreneurs, thus they are better able to cope with stereotype threat and become heathier. The following hypothesis is therefore proposed:

#### **H2**

 Among female entrepreneurs, firm age is positively related to their physical health.

**Fig. 1:**
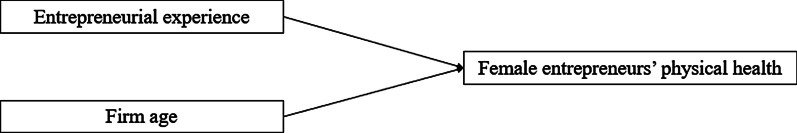
Theoretical framework

Thus, we showed the theoretical framework in Fig. 1.

## Methods

### Data collection and sample

We used the survey method to obtain data for Chinses entrepreneurs by questionnaire in Bohai Economic Rim [58, 59]. We collected our sample and data in Bohai Economic Rim, including Beijing, Tianjin, province of Shandong, Hebei and Liaoning. The reason to choose this area is that Bohai Economic Rim is one of the most developed areas for entrepreneurial activities in China, and new ventures are more concentrated in this area [58]. We surveyed via face-to-face interviews in these areas where there are many small businesses. We conducted the first survey in 2015 and obtained 1021 questionnaires. Three hundred twenty-one entrepreneurs in the area had not been contacted or were unwilling to participate in our second face-to-face investigation. We got 700 questionnaires back in the second survey in 2016. The distribution of the industries in the 700 samples is scattered. The percentage of ventures in the manufacturing industry is 67.43%, which is the highest. To ensure the validity of our research, we focused on the entrepreneurs who had founded a business in the manufacturing industry. Based on the industrial classification for national economic activities of the National Bureau of Statistics of China, the manufacturing industry refers to new products that have undergone physical or chemical changes. They are manufactured by machinery or by hand, and some products are manufactured in wholesale or retail. It includes the agricultural and sideline food processing industry, the food manufacturing industry, the textile industry, and so on.^4^ Besides, the development of manufacturing industry is important for local economics [60]. Thus, entrepreneurs in this field commonly face greater mental pressure, and they are suitable samples for an entrepreneurial health study. And then, we exclude the establishment of enterprise samples for more than 8 years to meet the characteristics of a start-up [61]. We also eliminate invalid samples with missing values, and obtained 300 samples. Finally, a female sample of 114 are selected from this 300 samples pool.

As shown in Table 1, the age of these female entrepreneurs is ranging from 28 to 55 years. Almost all of the participants completed high school or technical secondary school degrees. The samples are evenly distributed among the five cities and provinces in the Bohai Economic Rim. As for industry, the number of start-ups engaged in food manufacturing, clothing, shoe and hat industry and art products manufacturing is larger than those of start-ups in the other divisions of manufacturing industry. Additionally, the average number of previous startups of these female entrepreneurs is 0.72, and the average firm age is 5.16 years.

**Table 1:** Descriptive statistic

	Sample characteristics	Frequency		Sample characteristics	Frequency
Age	28–30	11	Region	Beijing	34
	31–40	48		Tianjin	26
	41–50	48		Hebei	16
	51–55	7		Shandong	24
Education	Middle school	3		Liaoning	14
	High school	36	Child	Have child	96
	Junior college	47		No child	18
	University	28	Industry	Food manufacturing	16
Marriage	Unmarried	6		Clothing, shoe and hat manufacturing	18
	Married	93		Art products manufacturing	10
	Divorce	15		Other manufacturing	70

	Mean value	Standard deviation	Median
Number of previous startups	0.720	0.507	1.00
Firm age	5.160	2.127	5.50
Total number of samples		114	

Data source calculated and sorted out by authors

### Measures

Independent variables: *Entrepreneurial experience.* The respondents’ entrepreneurial experience was primarily measured by the survey question, “How many times have you started a business before this start-up?” *Firm age.* This variable was assessed by deducting from 2016 the number of years since their enterprises were established.

Dependent variables: *Physical health.* The researchers used headaches as the most important variable to study the health of entrepreneurs. As an important indicator of personal health, headache is the most frequent complaint in people's daily lives [62], and it may cause more physical and mental health problems [63]. Therefore, headaches can be used as a representative indicator to measure the physical health of entrepreneurs. Among the respondents, those who had had a headache were regarded as unhealthy and were marked with a 0, and those who had not had a headache were regarded as healthy and marked with a 1.

Control variables: Entrepreneurs’ personal characteristics and the performance of start-ups might affect entrepreneurs’ health [7, 64], our research controlled for several individual and firm-level variables. In individual level, this paper controlled for the *age* of entrepreneurs and *educational attainment* [33]. The age of entrepreneurs was obtained by the number of years old and the educational attainment was obtained by the highest degree (primary school = 1, secondary high school = 2, senior high school/technical secondary school = 3, junior college = 4, undergraduate = 5, graduate = 6, Ph.D. = 7, other = 8). We also controlled for the *marital status* (married and divorced = 1; single = 0) and *child* [15]. The variable of child was measured by asking whether they had a child (yes = 1, no = 0). In addition, we controlled for the *lifestyle* of the participants [65]. The variable of lifestyle was measured by asking, “Do you do exercise frequently?” (yes = 1, no = 0). At the firm level, we controlled for *industry category*, *firm size* and *sales growth rate*. Based on industrial classification for national economic activities of the National Bureau of Statistics of China, there are thirties subdivisions of the manufacturing industry. We had controlled for the sub-industries in manufacturing. From the results of the descriptive statistics, we found that the samples in the food manufacturing industry, clothing, shoe, and hat manufacturing industry, and art products manufacturing industry are more than other sub-industries. We formed the dummy variables by defining whether the firms are in these three sub-industries. If yes, we coded as 1; if no, we coded as 0. We also formed a new dummy variable named other industries. It means the firms are not in neither of these three sub-industries. Firm size was measured by taking the natural logarithm of the total assets of the company in 2016. Sales was measured by asking the participants the average sales growth rate in 2016.

## Results

### Descriptive statistics and correlations

The descriptive statistics and the correlation coefficient matrix of the variables are shown in Table 2. The value of the variance inflation factors is all smaller than 2. It means that the results are not needed to consider the multicollinearity concerns [59].

**Table 2:** Correlation analysis

	Mean	SD	1	2	3	4	5	6
1. Entrepreneurs age	40.840	6.993	1.000					
2. Educational attainment	3.880	0.811	− 0.309**	1.000				
3. Marital status	0.820	0.389	− 0.053	− 0.016	1.000			
4. Child	0.840	0.366	− 0.127	0.113	0.167	1.000		
5. Exercise	0.740	0.442	0.001	0.230*	0.076	0.069	1.000	
6. Industry food manufacturing	0.140	0.349	− 0.016	− 0.032	− 0.134	0.106	0.127	1.000
7. Industry clothing, shoe and hat manufacturing	0.160	0.366	0.131	− 0.202*	0.082	0.056	0.040	− 0.175
8. Industry art products manufacturing	0.090	0.284	− 0.033	0.124	0.067	0.049	− 0.026	− 0.125
9. Firm size	5.436	1.447	0.148	0.121	− 0.040	0.099	0.100	0.007
10. Sales growth rate	14.680	10.762	0.068	0.115	0.020	0.160	0.031	0.156
11. Entrepreneurial experience	0.720	0.507	0.562**	− 0.343**	0.050	− 0.098	0.062	− 0.126
12. Firm age	5.160	2.127	0.179	0.078	− 0.018	0.055	0.176	0.161
13. Health status	0.816	0.389	0.038	− 0.128	0.066	− 0.082	− 0.078	− 0.069

	Mean	SD	7	8	9	10	11	12	13
1. Entrepreneurs age	40.840	6.993							
2. Educational attainment	3.880	0.811							
3. Marital status	0.820	0.389							
4. Child	0.840	0.366							
5. Exercise	0.740	0.442							
6. Industry food manufacturing	0.140	0.349							
7. Industry clothing, shoe and hat manufacturing	0.160	0.366	1.000						
8. Industry art products manufacturing	0.090	0.284	− 0.134	1.000					
9. Firm size	5.436	1.447	0.087	− 0.101	1.000				
10. Sales growth rate	14.680	10.762	− 0.012	− 0.118	0.599**	1.000			
11. Entrepreneurial experience	0.720	0.507	0.146	− 0.196*	0.110	− 0.046	1.000		
12. Firm age	5.160	2.127	− 0.010	0.006	0.359**	0.310**	0.000	1.000	
13. Health status	0.816	0.389	0.020	− 0.253**	− 0.032	− 0.092	0.319**	0.057	1.000

Data source calculated and sorted out by authors

*Means *p* < 0.05, ** means *p* < 0.01, two-tailed test

### Regression analysis

In this paper, we used logistic regression for the analysis. We ran the regression by using SPSS 26.0. The results are shown in Table 3. There was a Cox and Snell R^2^ increase from 0.093 (model 1) to 0.198 (model 2). The Nagelkerke R^2^ increased from 0.150 (model 1) to 0.323 (model 2). It suggests that the inclusion of the items of entrepreneurial experience and firm age greatly enhanced the explanatory degree of the models. The coefficient of the items of entrepreneurial experience and health status is positive and significant (model 2: b = 2.640, *p* = 0.002). The coefficient of the items of firm age and headache is also positive and significant (model 2: b = 0.271, *p* = 0.088). These results show that female entrepreneurs with more entrepreneurial experience are prone to be more health. When the firm age is larger, founders are prone to be more health. Hence, hypothesis 1 and hypothesis 2 are supported.

**Table 3:** Logistic regression results

Variable	Model 1	Model 2
Constant	3.429 (1.463)	6.287* (3.292)
Entrepreneur age	− 0.003 (0.007)	− 0.117** (4.290)
Educational attainment	− 0.262 (0.489)	− 0.168 (0.158)
Marital status	0.645 (0.997)	0.402 (0.308)
Child	− 0.573 (0.401)	− 0.428 (0.210)
Lifestyle	− 0.405 (0.368)	− 0.917 (1.505)
Industry food manufacturing	− 0.520 (0.495)	− 0.345 (0.156)
Industry clothing, shoes and hats manufacturing	− 0.400 (0.270)	− 0.697 (0.712)
Industry art products manufacturing	− 2.010*** (6.869)	− 1.736** (4.096)
Firm size	0.066 (0.087)	− 0.169 (0.443)
Sales growth rate	− 0.028 (0.840)	− 0.017 (0.253)
Entrepreneurial experience		2.640*** (9.820)
Firm age		0.271* (2.908)
Cox and Snell R-square	0.093	0.198
Nagelkerke R-square	0.150	0.323

The number in the parenthesis are the results from Wald-Test

Data source calculated and sorted out by authors

*Means *p* < 0.1, ** means *p* < 0.05, *** means *p* < 0.01, two-tailed test

### Robustness checks

To ensure the reliability of the conclusion, this study conducts one robustness tests by dividing entrepreneurial experience into two groups according to whether they have entrepreneurial experience (yes = 1, no = 0) and dividing firm ages into two groups according to median firm age, which is 5.50 years old: above or equal to 6 years old = 1 and under 6 years old = 0. Table 4 shows that the Cox and Snell R^2^ increases from 0. 093 (model 3) to 0. 212 (model 4). The Nagelkerke R^2^ increases from 0.150 (model 3) to 0.345 (model 4). This indicates that the items of entrepreneurial experience and firm age are contribute to the models. The coefficients of the entrepreneurial experience and health status is positive and significant (model 4: b = 2.864, *p* = 0.001). The coefficients of the firm age and health status is also positive and significant (model 4: b = 1.685 *p* = 0.027).

**Table 4:** Logistic regression results of robust test

Variable	Model 3	Model 4
Constant	3.429 (1.463)	7.739** (4.297)
Entrepreneur age	− 0.003 (0.007)	− 0.130** (4.785)
Educational attainment	− 0.262 (0.489)	− 0.163 (0.140)
Marital status	0.645 (0.997)	0.577 (0.591)
Child	− 0.573 (0.401)	− 0.253 (0.069)
Lifestyle	− 0.405 (0.368)	− 0.993 (1.720)
Industry food manufacturing	− 0.520 (0.495)	− 0.539 (0.375)
Industry clothing, shoes and hats manufacturing	− 0.400 (0.270)	− 0.935 (1.209)
Industry art products manufacturing	− 2.010*** (6.869)	− 2.065** (4.933)
Firm size	0.066 (0.087)	− 0.266 (1.007)
Sales growth rate	− 0.028 (0.840)	− 0.019 (0.304)
Entrepreneurial experience		2.864*** (10.394)
Firm age		1.685** (4.914)
Cox and Snell R-square	0.093	0.212
Nagelkerke R-square	0.150	0.345

The number in the parenthesis are the results from Wald-Test

Data source calculated and sorted out by authors

** means *p* < 0.05, *** means *p* < 0.01, two-tailed test

It seems that entrepreneurial experience and firm age are very robust variables. Hence, hypothesis 1 and hypothesis 2 of this paper are also supported. Thus, the results of this research are reliable and robust.

This paper also uses the male sample to verify the relationship between these two independent variables and health status (see Additional file 1). We find no similar results, so it can be proved that gender stereotype threat only exists in the group of female entrepreneurs.

## Conclusion and discussion

The Covid-19 pandemic leads small businesses to face a more uncertain business environment [3]. People might face unemployment stress [66] or infection-related stressors [67]. Female entrepreneurs are also faced with stereotype threat stress [40]. Female entrepreneurs should build a positive identity to get over the stereotype threat under the Covid-19 pandemic context. Based on stereotype threat theory, this paper used 2-year entrepreneurs’ tracking data from China’s Bohai Economic Rim to verify that the increase in entrepreneurial experience and the growth of a company’s age could help female entrepreneurs to maintain good health.

### Theoretical implications

The findings of the paper support the core argument that entrepreneurs’ experience and firm age will help female entrepreneurs maintain health. Previous research mainly examined how entrepreneurs’ personality (individual level) and entrepreneurial occupation (organizational level) influenced individuals’ health. However, less work had been conducted about how external dynamics factors affected female entrepreneurs’ health. Our research introduces two dynamic variables into the research of entrepreneur health: entrepreneurial experience (individual level) and firm age (organizational level). Our paper finds that these two variables can affect the health of female entrepreneurs, extending the research scope of previous studies.

In addition, this paper contributes to the study of female entrepreneurship. The previous psychological research had examined that stereotype threat did have a negative impact on individuals’ health [24–26]. During the process of entrepreneurship, female entrepreneurs are facing gender stereotype threat, while male entrepreneurs do not. However, women can reduce gender stereotype threat by establishing multiple positive personal identities [44] through entrepreneurial history. Using the sample of female entrepreneurs from China, this paper posits that increasing entrepreneurial experience and growing firm age may help women establish positive identity, which is an effective way to overcome gender stereotype threat, and thus may benefit to female entrepreneur’s health. This paper describes the factors affect the health of female entrepreneurs, enriching the research framework for female entrepreneurship.

### Practical implications

When people become entrepreneurs, they may endure greater stress than ordinary people. This could be worse for female entrepreneurs, as they may have to pay more efforts to balance work and family or to cope with stereotype threat in workplaces. Our paper suggests that persist in what they are doing may help female entrepreneurs maintain health. Social media can report more positive female entrepreneurs’ models and emphasize that females are qualified or even more suitable for entrepreneurship.

Secondly, this paper gives the practical implication from the policy perspective. This article finds that female entrepreneurs are more likely to be unhealthier in the art products manufacturing. Policymakers should increase paying attention to female entrepreneurs in art products manufacturing industry. We suggest that local government and business incubation centers appropriately organize training and exhibitions to help female entrepreneurs improve their entrepreneurial skills and broaden their visions. By helping female entrepreneurs to establish an experienced identity, it helps female entrepreneurs to keep healthy.

Additionally, the government should give female entrepreneurs more support in the early stage of entrepreneurship. Compared with mature companies, younger companies may encounter more difficulties. The government can help female entrepreneurs through the challenging early-stage by reducing taxes, reducing loan interest rates, and helping them obtain financing. the support of government policies can effectively limit the higher firm failure rate during the early years. A benign entrepreneurial environment is beneficial to female entrepreneurs to achieve their goals. Then, it helps them establish a successful identity, which improves the health of female entrepreneurs.

Lastly, we provide practical implications for governments and female entrepreneurs during the pandemic. Female entrepreneurs are suffering tremendous pressure in the pandemic. This hostile environment would challenge the health status of female entrepreneurs. On the one side of the results of this paper, the entrepreneurial experience could help female entrepreneurs keep healthy. The government should encourage female entrepreneurs who close their businesses forever during the Pandemic to reestablish a new one. On the other side of the results of this paper, firm age could help female entrepreneurs keep healthy. If the firms insist on opening during the pandemic, it might benefit female entrepreneurs’ health.

## Supplementary Information

**Additional file 1.** Logistic Regression Results for the sample of male entrepreneurs.

## Data Availability

The datasets used and/or analyzed during the current study are available from the corresponding author upon reasonable request.

## Footnotes

1 Danni He. (2015, October 10). Sohu News. Retrieved from https://www.sohu.com/a/34067945_114812.

2 Pengpai News. (2018, June 8). Retrieved from http://baijiahao.baidu.com/s?id=1602659781885961107&wfr=spider&for=pc.

3 Laura Didyk. Retrieved from https://baystbull.com/for-women-entrepreneurs-the-pandemic-is-hurting-business-and-their-mental-health/.

4 For more subdivisions of the manufacturing industry, please refer to the National Bureau of Statistics of China's regulations. http://www.stats.gov.cn/tjsj/tjbz/201301/t20130114_8675.html.
