# Supplementary material for: Does persistence make you healthy? An empirical study on female entrepreneurs from China
Source: BMC Womens Health. 2021 Sep 8;21:327. doi: 10.1186/s12905-021-01471-6 (PMC8425461; doi:10.1186/s12905-021-01471-6)
Supplement: Supplementary file 1 — Additional file 1. Logistic Regression Results for the sample of male entrepreneurs. [file 12905_2021_1471_MOESM1_ESM.docx]

Appendix Logistic Regression Results for the sample of male entrepreneurs.

| Variable | Model 5 | Model 6 |
| --- | --- | --- |
| Constant | 0.670 | 0.855 |
|  | (0.101) | (0.159) |
| Entrepreneur age | -0.001 | 0.007 |
|  | (0.001) | (0.058) |
| Educational attainment | 0.045 | 0.078 |
|  | (0.027) | (0.078) |
| Marital status | 0.528 | 0.427 |
|  | (0.836) | (0.542) |
| Child | 0.393 | 0.345 |
|  | (0.561) | (0.434) |
| Lifestyle | -0.327 | -0.377 |
|  | (0.514) | (0.662) |
| Industry food manufacturing | -0.735 | -0.604 |
|  | (1.840) | (1.189) |
| Industry clothing, shoes and hats manufacturing | 0.144 | 0.047 |
|  | (0.041) | (0.004) |
| Industry art products manufacturing | 0.648 | 0.813 |
|  | (0.924) | (1.417) |
| Firm size | -0.028 | 0.038 |
|  | (0.042) | (0.068) |
| Sales growth rate | 0.005 | 0.001 |
|  | (0.076) | (0.003) |
| Entrepreneurial experience |  | 0.058 |
|  |  | (0.015) |
| Firm age |  | -0.177* |
|  |  | (3.458) |
| Cox and Snell R-square | 0.039 | 0.057 |
| Nagelkerke R-square | 0.058 | 0.087 |

*Note:* * means *p* < 0.1, ** means *p* < 0.05, *** means *p* < 0.01, two-tailed test.

*Note:* The number in the parenthesis are the results from Wald-Test.

*Note:* Data source calculated and sorted out by authors.
